# Comparative study of left atrium epicardial fat tissue pattern using persistent homology approach

**DOI:** 10.1186/s13104-022-06173-2

**Published:** 2022-09-15

**Authors:** Deepa Deepa, Yashbir Singh, Wathiq Mansoor, Weichih Hu, Rahul Paul, Gunnar E. Carlsson

**Affiliations:** 1 Biomedical Engineering, Chung Yuan Christian University, Zhongli, Taiwan; 2 Department of Radiology, Mayo clinic, Rochester, MN USA; 3 Engineering & Information Technology, University of Dubai, Dubai, UAE; 4 Department of Radiation Oncology, Massachusetts General Hospital, Boston, MA USA; 5 Department of Mathmetics, Stanford University, Stanford, CA USA

**Keywords:** Atrial fibrillation, Barcode, Persistent homology, Pixel value masking, Topology

## Abstract

**Objective:**

Atrial Fibrillation (A-fib) is an abnormal heartbeat condition in which the heart races and beats in an uncontrollable way. It is observed that the presence of increased epicardial fat/fatty tissue in the atrium can lead to A-fib. Persistent homology using topological features can be used to recapitulate enormous amounts of spatially complicated medical data into a visual code to identify a specific pattern of epicardial fat tissue with non-fat tissue. Our aim is to evaluate the topological pattern of left atrium epicardial fat tissue with non-fat tissue.

**Results:**

A topological data analysis approach was acquired to study the imaging pattern between the left atrium epicardial fat tissue and non-fat tissue patches. The patches of eight patients from CT images of the left atrium heart were used and categorized into “left atrium epicardial fat tissue” and “non-fat tissue” groups. The features that distinguish the “epicardial fat tissue” and “non-fat tissue” groups are extracted using persistent homology (PH). Our result reveals that our proposed research can discriminate between left atrium epicardial fat tissue and non-fat tissue. Specifically, the range of Betti numbers in the epicardial tissue is smaller (0–30) than the non-fat tissue (0–100), indicating that non-fat tissue has good topology.

## Introduction

Atrial fibrillation (A-fib) is a heart disorder characterized by an abnormal heart beat in which the heart races and beats in an unpredictable way. It is seen that A-fib results in the development of blood clots in the upper chamber of the heart which later flow to different organs and confine the blood supply to tissues and elicit heart attacks. It is the most prevalent type of arrhythmia, and causes a higher mortality rate worldwide. It is assumed the mortality and morbidity related to A-fib can significantly increase up to three-fold within the next 30 years [1–3]. The presence of elevated epicardial fat exists in patients with permanent A-fib [4]. The epicardial fat is found on the top of the left atrium close to the left atrial appendage and lateral to the mitral isthmus and is more in the superior half of the left atrium than in the inferior half [4, 5]. The peri-atrial epicardial fat may contribute to the formation of A-fib by obstructing the flow of electrical signals to all regions of the heart [4]. Here in this study, our main concern will be on the epicardial fat present in the left atrium. In past few decades, medical imaging data has been piling up in abundance. Medical imaging provides tremendously meaningful information which can be used to extract patterns and can be saved for future reference. With the recent up gradation of computational methods, researchers have offered a variety of concepts and tools that may help to recognize a specific disease pattern through topological features. Topological Data Analysis (TDA) gives a fresh perspective in this field to analyze the datasets using topological approaches. The fundamental goal of TDA is to build tools for studying qualitative characteristics of data using ideas and results from geometry and topology [6]. PH is one of the TDA approaches for detecting topological features of data. This method is based on algebraic computation, which provides an effective theoretical framework for understand qualitative characteristics of data with complex structures [6]. Here, we introduce a method that allows the extraction of topological features that differentiate “left atrium epicardial fat tissue” and “non-fat tissue” group patients.

## Main text

### Methods

#### Data information and preparation

This work included eight male patients with A-fib in 52–61 age groups. Our work was based on delayed enhancement cardiac CT image datasets obtained by ten sets of time frames at the same position using different contrast mediums, each containing one complete cardiac cycle. Philips computerized tomography instrument was utilized to obtain the image dataset of. Each patient's data set included 392 images with 512 × 512 dimensions that covered the entire thoracic region.

**Fig. 1:**
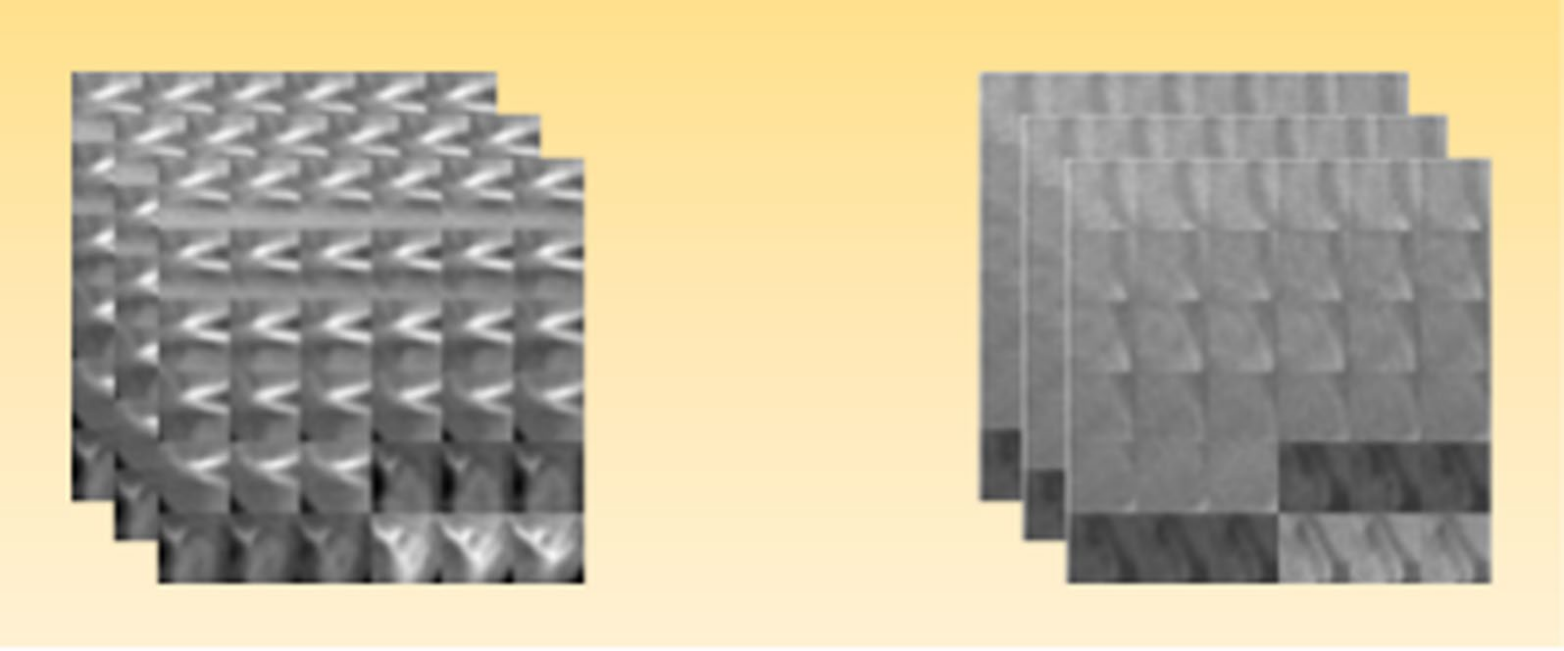
(i) Patch representation of epicardial fat tissue. (ii) Patch representation of Non-fat tissue

All the 392 images were stacked up to get a 3D view of the thoracic region. Then we extracted only those images which included only the heart. These images were 204 in number. Since the thoracic region contains the heart, ribcage and spinal cord, which can make difficult the visualization of the heart, that is the rationale for our clearing out the surrounding structures by removing the pixels representing these structures. The 3D view was visualized through the Volume Viewer App of MATLAB R2018a. After that, a masking filter was applied to these images to highlight the required area by changing the color of the original images. An image display threshold setting of –190 to –30 Hounsfield Units (HU) was identified for epicardial fat on grayscale; other research also suggested a threshold of -200 to -50 HU [5, 7]. The images were sent to mask the pixel values which lie between -190 to -30 HU. The fat tissues on the atrium of the heart were identified using the pixel value range masked over the entire dataset. The above-mentioned methodology has already been described in our previously published study [8]. We extracted the desired dataset of 36 images from each patient’s data which contains the fat region of the atrium. We cropped these fat regions from all the images and made 36 patches of 32 × 32 dimensions. These 36 patches were concatenated to form one large patch. Additionally another 36 patches are extracted from the atrium containing non-fat tissues and preceded the same way as mentioned (Fig. 1).

#### Persistent homology filtration

Key objects in topological data analysis are filtered simplicial complexes, called filtrations. Homology is a method for in an accurate measuring the shape of a geometric object by counting holes or features of various dimensions. Its output is a vector space for each non-negative integer k whose dimension (called the k-th Betti number) is the feature count for holes of dimension k. This notion extends to filtered simplicial complex by producing a “persistence vector space”, the analogue of vector spaces, as well as the analogue of dimension, which is called a persistence barcode or persistence diagram. These PH diagrams can be thought of as finite collections (unordered) of intervals, and one uses various algebraic combinations of the lengths of the intervals as well as their midpoints [9].

**Fig. 2:**
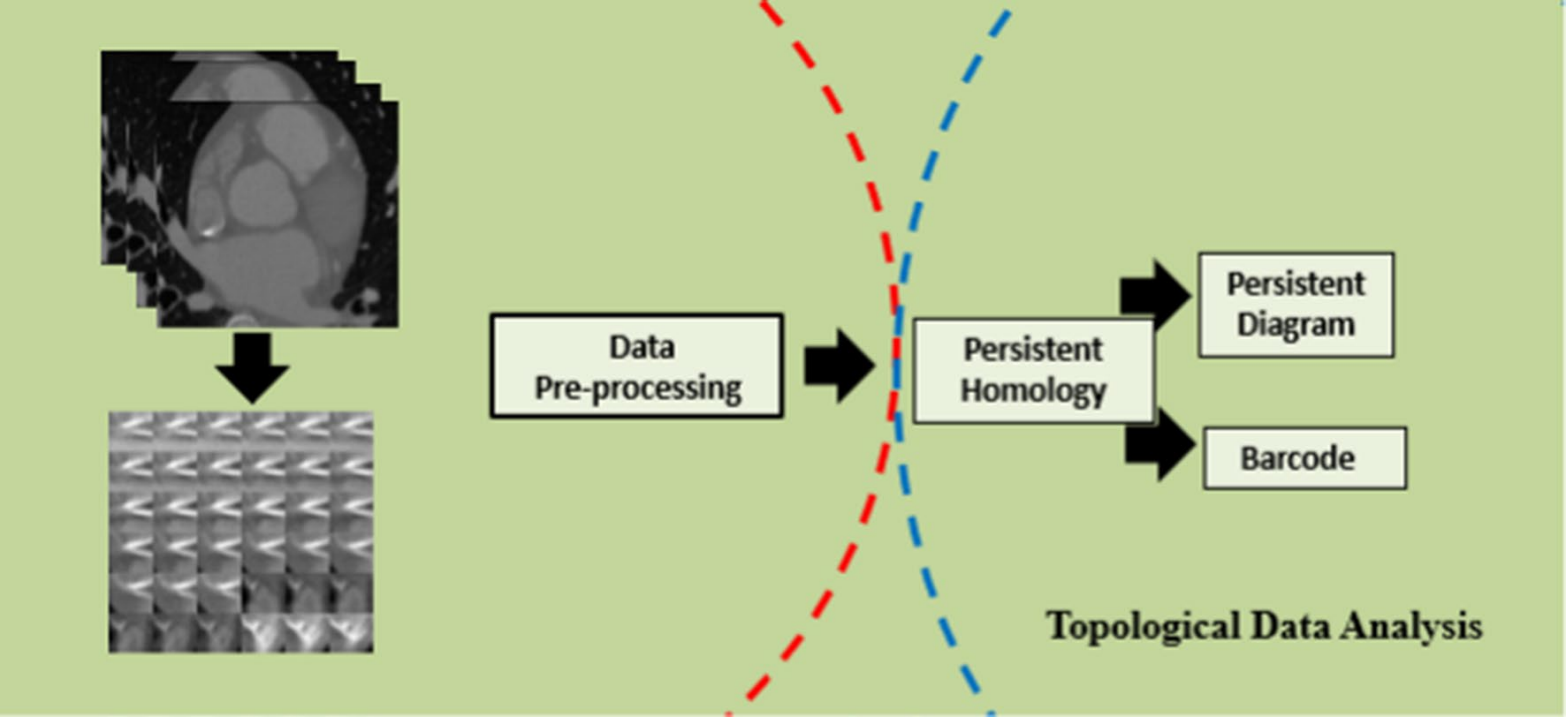
Topological data analysis workflow with imaging signal input

Filtration is the first step of PH which produces a series of simplicial complexes for a scale proximity parameters (ε). We observe the filtration procedure for a point cloud data set where each point is surrounded by a sphere of radius ε. We draw an edge between two spots at each intersection of two spheres. Filtering data creates a simplicial complex space from which PH quantifies the presence of n-dimensional holes, which include 0-dimensional holes, 1-dimensional holes, circles/loops/tunnels, and 2-dimensional holes. Since the best value for the scale ε cannot be determined, the primary principle of PH is to move through all possible values (0) to see how the homology of these components changes [10]. We evaluate the times of birth (ε emerges) and death (ε vanishes) for each n-dimensional structure (Fig. 2).

**Fig. 3:**
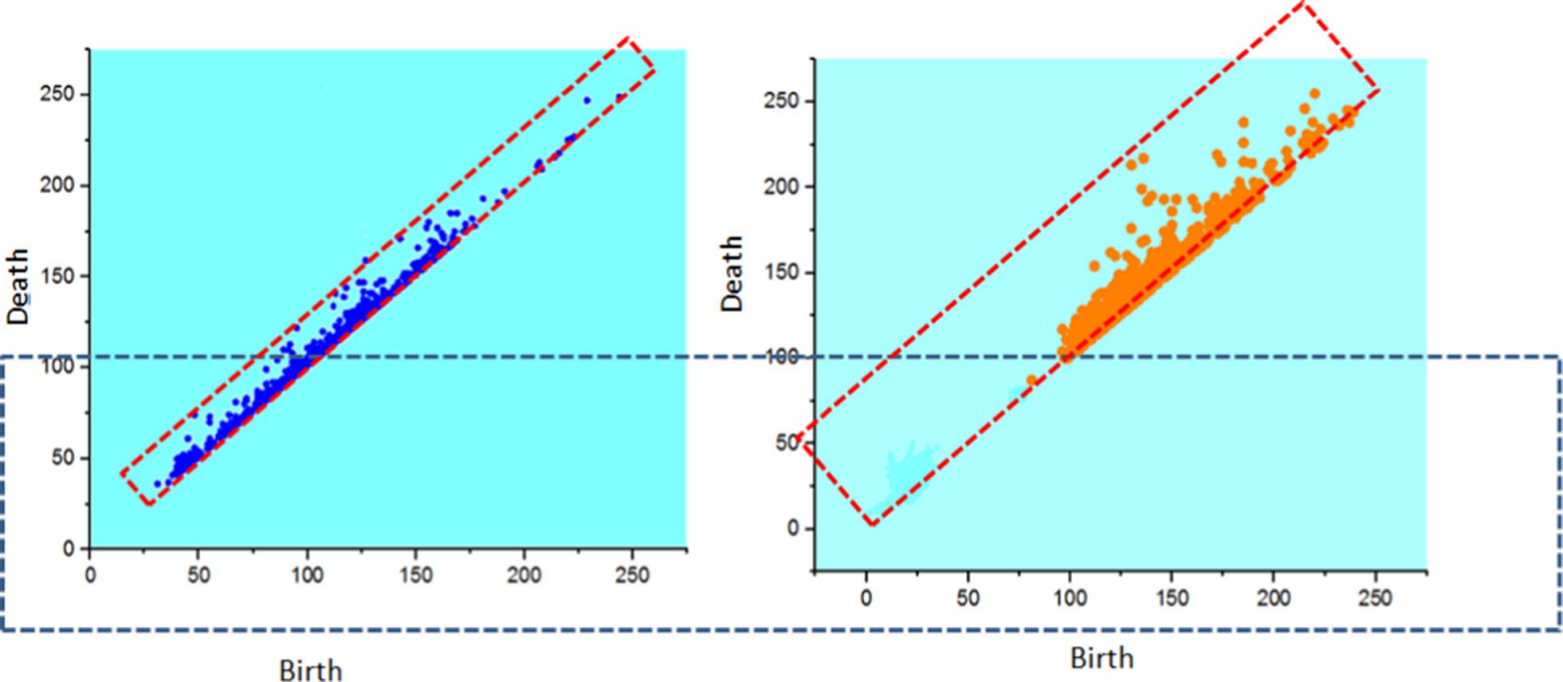
(i) PH of epicardial fat tissue. (ii) PH of Non-fat tissue.

### Results and discussion

In left atrium epicardial fat tissue, we see the range of Betti numbers varies less (0–30), while in the non-fat tissue, the range of the Betti numbers is large (0–100). After Betti number, we plotted PH. Figure 3 shows the PH diagrams of left atrium epicardial fat and non-fat tissue. In the diagram of epicardial fat tissue, the points are closer towards the diagonal line, which means a topology with many small holes, but in non-fat tissue, the points are more scattered and concentrated away from diagonal that means topology is smoother on a small scale. This is the other way to calculate the pattern between two groups of tissues.

Fat tissues are the adipose tissue that helps in storing energy, and in distress, they provide support to the system for the proper execution of body functions. They insulate the body and act as an endocrine organ. The adipose tissue between the visceral pericardium and the myocardium is known as epicardial fat [11]. The increased epicardial fat on the left atrium wall hampers electrical conduction. Several studies have been reported that provide evidence of epicardial fat in the atrium causing A-fib [12, 13]. To measure cardiac fats, a couple of imaging modalities such as magnetic resonance imaging (MRI), echocardiography, and computed tomography (CT) are currently accessible in the market. However, detection of the fat can need either manual strategies which can be tedious or using some available strategies [14–17]. TDA combines algebraic topology and statistical learning techniques to provide a mathematical foundation for studying the shape of data. TDA also offers dimensionality reduction and noise stability. Here, we applied PH, an algebraic method of TDA that discover the topology of data to find new and distinctive features. The basic idea behind PH is to replace data points with a parametrized family of simplicial complexes, which can generally be depicted as a union of points, edges, triangles, tetrahedrons, and higher-dimensional polytopes, and encode the change of the simplicial complexes' topological features (such as the number of connected components, holes, and voids) across various parameters for data analysis [18–20]. Our PH diagrams represent the distinct comparison between fat tissue and non-fat tissues in our data. Our results have shown that this method may help in identifying the fat tissue from non-fat tissue for better stratification. In conclusion, by analyzing CT patches using a topological data analysis approach known as persistent homology, we have identified patterns in barcodes and persistence diagrams that discriminate cardiac patients whoexperience epicardial fat tissue versus those who do not have epicardial fat tissue. While promising, our results will need to be validated on a larger cohort.

### Limitations of the study

We have used very small number of the patients with A-fib. The data was validated under the supervision of cardiologists.

## Data Availability

The dataset of the current study is available from the first author on reasonable request.

## Abbreviations

A-fib: Atrial Fibrillation
PH: Persistent homology
TDA: Topological Data Analysis
HU: Hounsfield Units
